# In Vitro Simulation of Dental Implant Bridges Removal: Influence of Luting Agent and Abutments Geometry on Retrievability

**DOI:** 10.3390/ma13122797

**Published:** 2020-06-21

**Authors:** Andrea T. Lugas, Mara Terzini, Elisabetta M. Zanetti, Gianmario Schierano, Carlo Manzella, Domenico Baldi, Cristina Bignardi, Alberto L. Audenino

**Affiliations:** 1 Department of Mechanical and Aerospace Engineering, Polytechnic University of Turin, 10129 Turin, Italy; andrea.lugas@polito.it (A.T.L.); cristina.bignardi@polito.it (C.B.); alberto.audenino@polito.it (A.L.A.); 2 Polito^BIO^Med Lab, Polytechnic University of Turin, 10129 Turin, Italy; 3 Department of Engineering, University of Perugia, 06125 Perugia, Italy; elisabetta.zanetti@unipg.it; 4 Department of Surgical Science, C.I.R. Dental School, University of Turin, 10126 Turin, Italy; gianmario.schierano@unito.it (G.S.); carlo.manzella@gmail.com (C.M.); 5 Division of Prosthetic Dentistry, Department of Surgical Sciences (DISC), University of Genoa, 16132 Genoa, Italy; baldi.domenico@unige.it

**Keywords:** dental bridge, abutments geometry, luting agents, retrieval, Coronaflex

## Abstract

Implant fixed dental prostheses are widely used for the treatment of edentulism, often preferred over the screw-retained ones. However, one of the main features of an implant-supported prosthesis is retrievability, which could be necessary in the case of implant complications. In this study, the retrievability of implant-fixed dental prostheses was investigated considering two of the main factors dental practitioners have to deal with: the abutments geometry and the luting agent. Impulsive forces were applied to dental bridge models to simulate crowns’ retrievability in clinical conditions. The number of impulses and the impulsive force delivered during each test were recorded and used as retrievability indexes. One-hundred-and-five tests were conducted on 21 combinations of bridges and luting agents, and a Kruskal-Wallis test was performed on the results. The abutment geometry significantly influenced the number of impulses needed for retrieval (*p* < 0.05), and a cement-dependent trend was observed as well. On the other hand, the forces measured during tests showed no clear correlation with bridge retrievability. The best retrievability was obtained with long, slightly tapered abutments and a temporary luting agent.

## 1. Introduction

Implant-fixed dental prosthetic reconstruction is an established treatment for edentulous patients, and it became the standard of care in dental medicine due to the progress in technologies related to osseointegration [1,2]. The prostheses can be screw- or cement-retained, and nowadays, there is no evidence on the superiority of one technique over the other [3]. However, in some cases, the choice is forced by the patient’s conditions, and a screw- or cement-retention might be needed. In the recent past, cement-retained fixed partial dentures (FPDs) gained popularity due to their aesthetic results, ease of fabrication, and passivity of fit [4,5,6,7,8]. However, cementation disadvantages have been highlighted in the literature [2,9], one of which is the difficulty in implant retrieval. In FPDs, sometimes biological, mechanical, and aesthetic problems may occur [10] and the retrieval of the dental crown might be necessary. Ideally, the cementation should be strong enough to bear masticatory loads avoiding cement failure and weak enough to allow easy and safe removal in case of need [9]. Luting agent choice is one of the main factors in crown retrievability [11,12,13]. For this reason, temporary cements (e.g., zinc-oxide eugenol) are often preferred over permanent ones (e.g., zinc-phosphate) to ensure retrievability [14,15,16]. Other factors influencing crown retention are abutment size and geometry. In particular, several studies in literature highlight a stronger retention with high abutments over short ones, and a loss of retention when the taper angle is increased [17,18,19]. These three main factors (abutment height, taper angle, and luting agent) were largely investigated in previous studies, both with uniaxial tensile tests [19,20,21] and impulsive loads application [22,23,24,25]. However, to the authors’ knowledge, all the data provided in literature derive from tests on single implants. The present study aims to simulate reality more closely compared to our previous work [22], in which the data were collected from tests on single implants: similar tests are now performed on dental bridges (i.e., multiple implant-supported restorations) models. The influence of abutments geometry and three different luting agents on bridge retrievability through impulsive loads was investigated, using the number of performed impulses and the forces generated during the process, as retrievability indexes.

## 2. Materials and Methods 

### 2.1. Specimen Preparation

**Figure 1:**
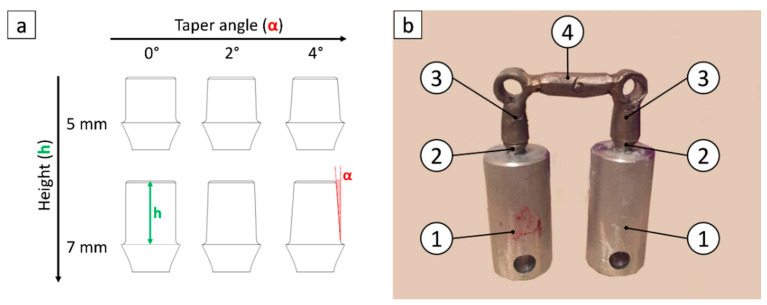
(**a**) Abutments geometry. (**b**) Dental bridge model: (**1**) aluminum supports, (**2**) titanium abutments, (**3**) Wegold N2 copings, (**4**) Pagalin 2 bar.

Seven different three-elements dental bridge models (referred to as bridges in the following) were used in this study. Each bridge included two titanium abutments and two Wegold N2 alloy (Wegold Edelmetalle GmbH, Wendelstein, Germany) copings realized as described in a previous work [19]. The fit between each abutment and its coping was evaluated with a silicon disclosing medium, using a loupe system. Copings were equipped with a ring in their upper part to ensure a gripping point for an alternative removal, in the event that removal during the test was not successful. Seven Pagalin 2 alloy (Cendres+Métaux SA, Biel/Bienne, Switzerland) bars were laser-welded with a coping on each end, thus obtaining seven bridges. Fourteen abutments, with a height ranging between 5 mm and 7 mm and a taper angle ranging between 0° and 4° (Figure 1a), were screwed onto 14 titanium implants (Brånemark System Mk III TiUnite RP, diameter = 3.75 mm, length = 13 mm, Nobel Biocare Italiana Srl, Milan, Italy), which were in turn screwed onto 14 custom-manufactured aluminum cylindrical supports (diameter = 15 mm, length = 25 mm) designed with SolidWorks^®^ CAD (Dassault Systemes, Waltham, MA, USA). Each coping was then cemented onto the corresponding abutment (Figure 1b).

Three different luting agents were used for coping-abutment cementation during this study: a temporary, self-curing zinc-oxide non-eugenol cement (Temp Bond NE, Kerr Italia, Salerno, Italy), a composite, self-curing cement for temporary aesthetic cementations (Telio CS Link, Ivoclar Vivadent, Leicester, UK) and a definitive zinc-phosphate cement (Harvard Cement, Harvard Dental Company, Hoppegarten, Germany). All cements were prepared in accordance with the manufacturers’ instructions in a dental office. Table 1 lists the bridge models obtained.

### 2.2. Test Setup

**Figure 2:**
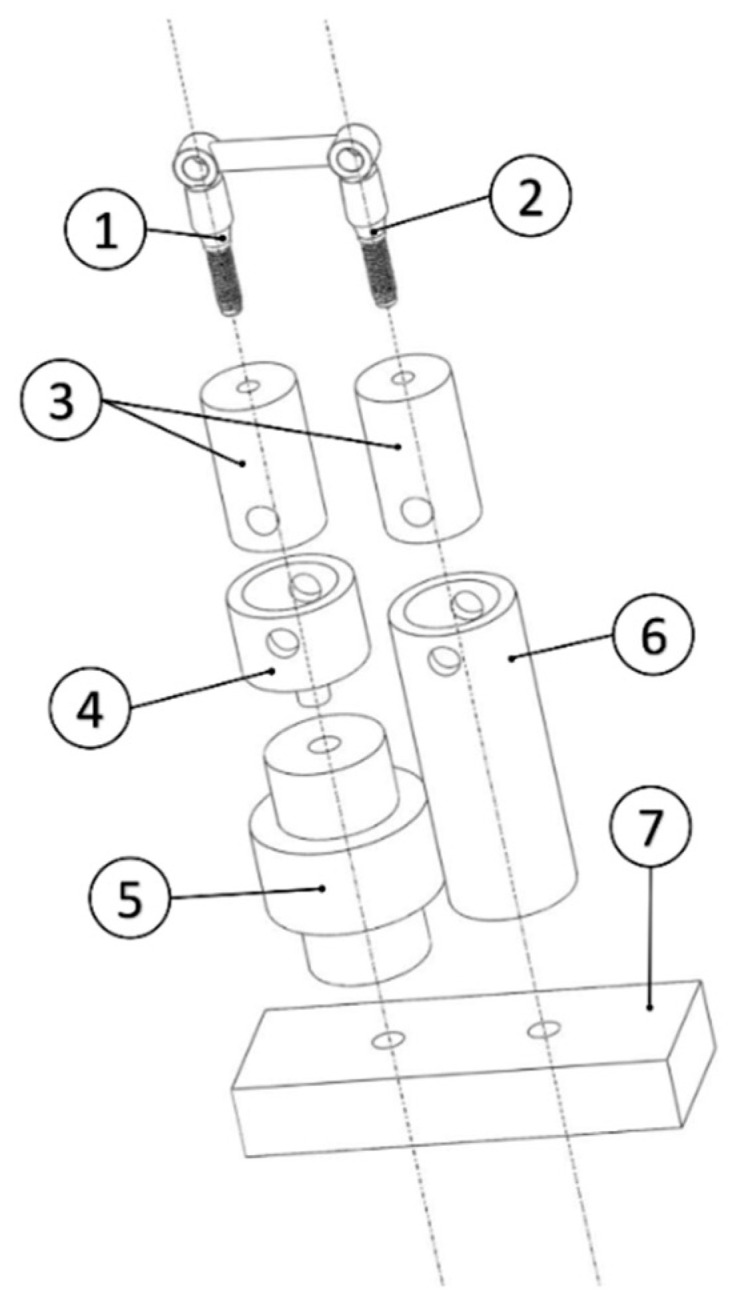
Specimens support: (**1**) the most retentive abutment (i.e., abutment 1 in Table 1), (**2**) the least retentive abutment (i.e., abutment 2 in Table 1), (**3**) implants holders, (**4**) connection between the holder of the most retentive abutment and the load cell, (**5**) load cell, (**6**) connection between the other implant holder and the base, (**7**) rectangular base.

The data acquisition system was described in our previous study [22]. It is composed of a load cell, which was positioned under the most retentive abutment of the bridge (i.e., the one with the lowest taper angle, according to the results of the previous study [22]) during each test, an amplifier, and a data acquisition board. The acquisition frequency was set at 51.2 kHz and signal acquisition was performed by means of LabVIEW SignalExpres. In order to allow a precise and repeatable connection of the specimens with the test bench, a series of supports were designed with SolidWorks and linked together with screws and linchpins. The aforementioned aluminum cylindrical supports were matched before each test with two holders: one was screwed onto the load cell, which was in turn connected with an aluminum base, and the other was directly linked with the base (Figure 2). The threaded holes in the rectangular base are positioned at a distance of 25 mm from each other. The base was secured in a vice throughout the tests.

### 2.3. Test Protocol

**Figure 3:**
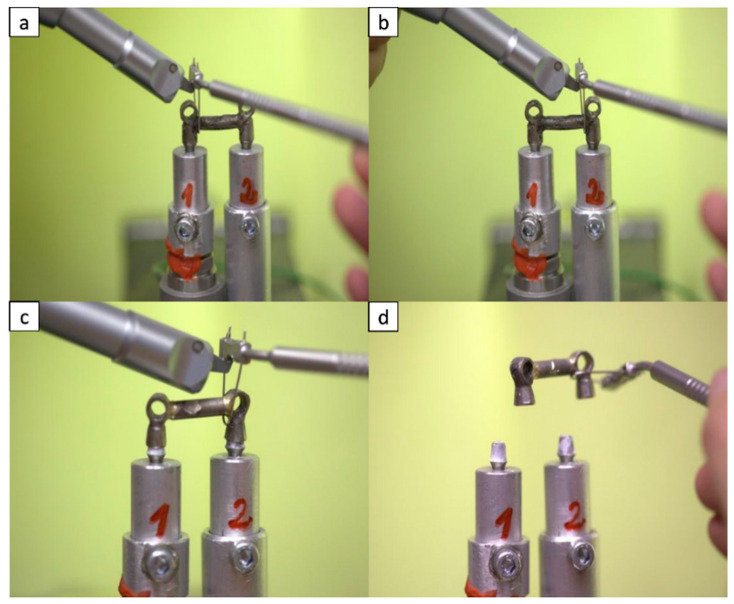
Test protocol: (**a**) impulses delivered near to the most retentive abutment, (**b**) impulses delivered at the other abutment, (**c**) cement fracture, (**d**) complete bridge removal.

After the cementation, the specimens were left to rest for at least 24 h before the test. The retrieval of the bridges was performed using Coronaflex^®^ (KaVo Dental Excellence, Biberach/Riß, Germany). Impulse force and operating pressure of the extraction tool were set to 400 N and 4 bar respectively. The impulsive forces were applied to the implants using a loop and a loop holder, provided with the extraction tool. The loop was placed under one end of the bridge (Figure 3a), and up to 15 impulses were delivered with Coronaflex^®^ on the loop holder. Then, the loop was moved to the other end of the bridge (Figure 3b) and the procedure was repeated. A maximum of 60 impulses (four series of 15 impulses) were delivered on each bridge. If there was observable cement fracture before the 15th impulse at the stressed site, the loop was moved to the other side; if there was cement fracture at the non-stressed site, additional impulses were delivered at the undamaged end of the bridge until a maximum of 30 impulses. The impulses always started at the most retentive abutment (referred to as abutment 1 in Table 1).

The total number of impulses applied during each test and the force trend over time were recorded. Bridges with one coping or both copings not detached at the end of the test were considered not removable. After each test, the copings were cleaned from the residual cement by hand and with an ultrasonic bath in a cement solvent. Five removal attempts for each bridge-cement combination were conducted, performing a total of 105 tests (7 bridges × 3 luting agents × 5 replicas).

### 2.4. Data Analysis

The number of impulses and the peak force during each impulse were computed from forces trends over time with a custom script in MATLAB 2019b (MathWorks, Inc., Natick, MA, USA). The total number of impulses and the average force (i.e., the mean of the force peak values during the impulses) were selected as retrievability indexes. A Kruskal-Wallis test was used to investigate the influence of (1) bridge (i.e., abutments geometry) and (2) luting agent on both indexes. A Bonferroni post hoc test was also performed on the impulses number to verify the inter-bridge comparison. 

## 3. Results

**Figure 4:**
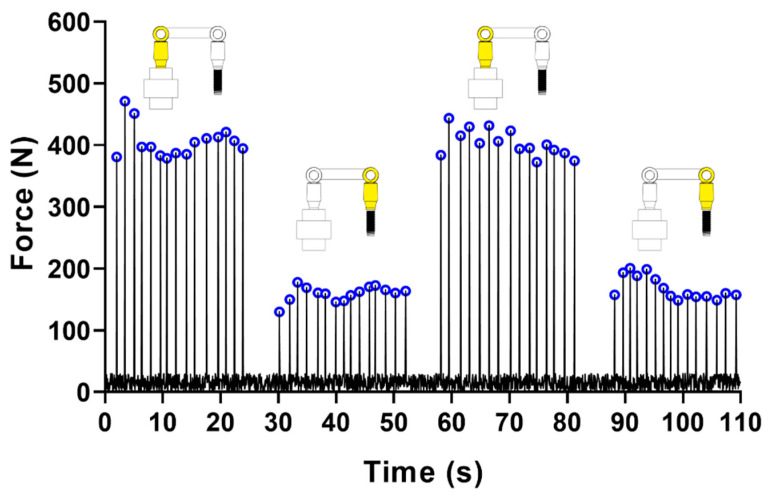
Representative force trend over time: detected force peaks are encircled in blue. The stressed side of the bridge during each series of impulses is highlighted in yellow. The load cell drawing was added for representative purposes, the scales of the cell and the bridge are not consistent.

Figure 4 shows a representative force trend over time recorded for a non-retrieved bridge. As previously pointed out, four series of 15 impulses were delivered on the bridge, resulting in four series of peaks, of variable magnitude depending on the delivery location. The average of the measured peak forces was considered as an indicator of the entire bridge retention. 

**Figure 5:**
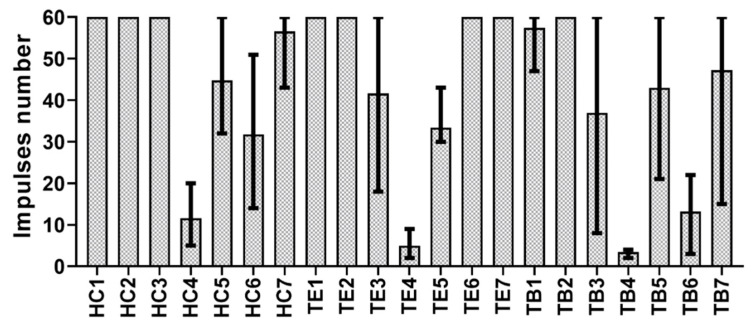
Number of impulses needed for retrieval with each luting agent-bridge combination.

The 105 tests performed were divided into 21 subgroups according to the different combinations of the two variability factors (3 luting agents × 7 bridges). Each group will be described in the following with two letters representing the luting agent (HC for Harvard Cement, TE for Telio CS Link and TB for Temp Bond) and a whole number ranging between 1 and 7 (for bridges 1 to 7, corresponding to abutments geometry shown in Table 1). The average values of the impulses number, as well as the upper and lower limits, are shown in Figure 5. Bridges 1 and 2 were the most retentive regardless of the luting agent, while bridge 4 needed the lowest number of impulses to be removed. Bridge number 1, indeed, was only removed in one case, when cemented with Temp Bond. In all other tests on bridges 1 and 2 cemented with Harvard Cement and Telio CS Link, the maximum number of impulses was reached with no removal of the copings.

**Figure 6:**
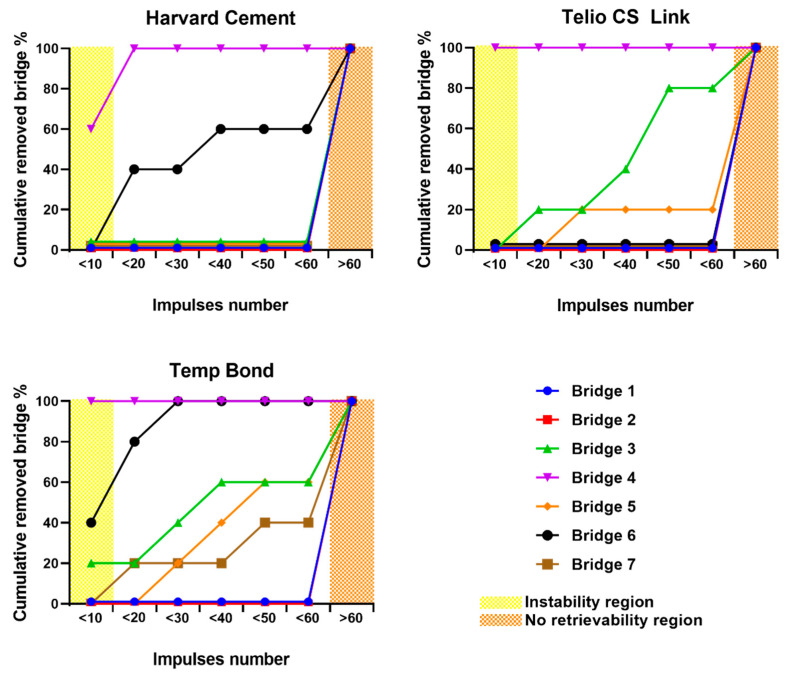
Removed bridges percentage vs impulses number. Each data point represents the total percentage of tests during which the bridge was removed within the corresponding number of impulses in the abscissa axis.

Moreover, the number of impulses was computed as follows: cementations fractured with 10 or fewer impulses were considered too weak, combinations which needed more than 10 and up to 60 impulses for the complete removal of both copings were considered optimal for retrievability, while bridges with non-removed copings were considered not suitable for retrievability (Figure 6). 

The combination with the best result (i.e., the highest percentage of copings removed within the optimal range of impulses number) was bridge 3, which has two identical abutments, with height = 7 mm and taper angle = 2°, cemented with Telio CS Link, a temporary cement. On this combination 80% of tests performed provided a complete removal of the bridge within the optimal range of 11 to 60 impulses.

**Figure 7:**
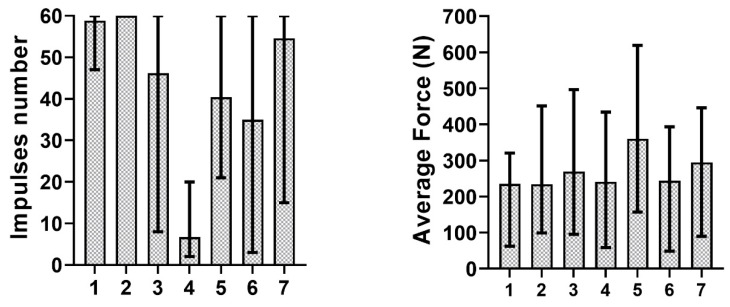
Bridge influence on impulses number and average force.

**Figure 8:**
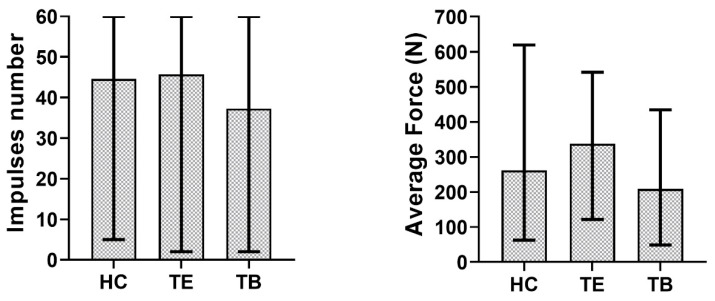
Cement influence on impulses number and average force.

In order to observe the influence of the two factors separately, the mean and the range of variability of delivered impulses number and measured forces were computed among the three different luting agents and the seven different bridges. The results are shown in Figure 7 and Figure 8.

The Kruskal–Wallis test confirmed the statistically significant influence of the abutments geometry on the impulses needed for bridge removal and the influence of the luting agent choice on the forces generated during the tests (*p* < 0.05). However, there was no statistical significance of the influence of types of cement on the impulses number and of the abutments geometry on the force. A Bonferroni correction was used on the data in order to compare the influence of the different bridges on the copings retrievability. The number of impulses performed on bridge 4, regardless of the cement, was significantly different from the impulses applied on the other bridges. Moreover, bridge 2 behavior was significantly different, other than bridge 4, from bridges 5 and 6.

## 4. Discussion

Most of the literature data on dental crown retrievability is related to the uniaxial force needed for retrieval, obtained through quasi-static tensile tests [26,27,28]. However, in clinical practice, impulsive loads are applied to remove crowns when needed [14], and the number of impulses is taken into consideration by clinicians to evaluate retrievability [29]. In this study, Coronaflex^®^ was used to simulate the clinical removal of implant fixed dental prostheses. This tool is currently used in clinical practice and its efficiency and repeatability among different operators have been demonstrated [24,25]. During this work, both the impulses number and the impulsive forces were used as retrievability indexes. Figure 5 shows the distribution of the number of impulses applied to the implants for each luting agent-bridge combination. Bridges 1 and 2 are the only ones with non-tapered abutments on both ends, and the results are in accordance with the established relationship between the abutment taper angle and the coping retrievability. Indeed, it is well-known, from both experimental [30,31,32] and clinical [29] studies, that the abutment retentiveness increases with its height and decreases with its taper angle. On the other hand, bridge 4 was the only one with two 5 mm high and tapered abutments (i.e., a poorly retentive geometry), and it behaved, as expected, as the least retentive one with all cements.

Figure 6 shows bridge retrieval suitability for each combination of abutment geometry and dental cement. Bridge 3 cemented with Telio CS Link was the best combination. This result is consistent with the fact that Bridge 3 has an average-retentive geometry for both copings, ensuring stability without compromising retrieval. Most bridges were successfully retrieved also with Harvard-cemented bridge 6 and Temp Bond-cemented bridge 5. However, bridge 5 resulted not advisable for retrievability nor adequate retention, because it has a very retentive cylindrical abutment (height = 5 mm, taper angle = 0°) and a very weak one (height = 5 mm, taper angle = 4°). This caused, in a few cases, the detachment of the least retentive coping within 5 impulses or less, which is an index of instability, while the most retentive coping was not removed within 30 impulses. Moreover, unexpected results were obtained from bridge 6, since it resulted to be more retrievable when cemented with Harvard Cement, which is a permanent cement, than with Telio CS Link, which is a temporary one. This aspect can be observed in Figure 6, as bridge 6 resulted not removable when it was cemented with Telio CS Link, while it was removed in the 60% of tests when cemented with Harvard Cement. Cementation of bridge 4 was too weak regardless of the cement used, while the other combinations (related to bridge 1, 2, and 7) resulted not suitable for retrievability.

Figure 7 and Figure 8 show the influence of cement and abutment geometry separately. It can be observed that the most retentive bridges were those with two cylindrical (i.e., not tapered) abutments, which agrees, as previously pointed out, with literature evidence. Moreover, bridge 3 (with two abutments which have 7 mm of height and 2° of taper angle), was less retentive than bridge 1 (which have shorter but not tapered abutments). These aspects highlight a stronger influence of the taper angle rather than the abutment height. Regarding the three different luting agents, Figure 8 shows that Harvard Cement and Telio CS Link provided cementations with a similar number of impulses needed for bridge removal, while Temp Bond was less retentive, and this aspect reflects previous findings. Indeed, zinc-oxide non-eugenol cements, like Temp Bond NE, are temporary cements with low compression and tensile strength and inherent brittleness [33]. In more detail, Harvard Cement resulted more retentive than Telio CS Link according to tests on almost all bridges. This result is in accordance with clinical evidence. Harvard Cement is indeed a zinc phosphate luting agent, and these cements are considered a good choice for luting metals, because of their strength, physical properties, and lack of technique sensitivity [34]. Therefore, the similarity between the average behavior of the first two luting agents may have been determined by the unexpected results on bridge 6. The use of adhesive cements would probably have provided more retentive solutions and allowed the difference between a temporary and a permanent cementation to be underlined. However, these types of cement are not recommended for retrievable restorations because of the difficulty of their removal from the implant [35]. Both in Figure 7 and Figure 8, the mean force during removal does not appear to be related to the impulses number nor with the cement-bridge combination. This result is in agreement with our previous work [22], in which a strong dependence of the number of impulses on the aforementioned indexes was established, but the same dependence was not steadily observed for the force values. The authors of [36] obtained similar results. Indeed, they did not find a significant difference in the number of impulses between different types of cement, and the number of impulses and the tensile forces were not always correlated. However, our results about force values could have been affected by the test setup. Indeed, the force measured under only one of the two abutments was considered as an index of the total force borne by the bridge. In addition, in order to obtain a more repeatable testing method and to avoid the introduction of more variability factors, no aging was performed during this study. This could have influenced our results as well, aging being a significant factor in dental prostheses retentiveness [37].

The statistical analysis highlighted the significance of the different impulses number needed to retrieve different bridges. Bridge 4 behaved in a significantly different way compared to all other cements. This can be related to the fact that bridge 4 has the least retentive couple of abutments, being both 5 mm long and with a taper angle of 2° on one side and 4° on the other. Moreover, bridge 2 has the most retentive geometry (i.e., two abutments with height = 7 mm and cylindrical shape), and its difference with the three least retentive bridges, i.e., bridges 4, 5 and 6, resulted significant.

## 5. Conclusions

This study was designed as an upgrade of our previous work on single implants. The results obtained are consistent regarding the impulses number and the abutment geometry. However, no significance was established for the relation between the luting agent and the impulses number. The different bridge-luting agent combinations did not affect the forces generated during the bridge removal with a steady trend, as happened with the single abutment-coping coupling. These results highlight the differences between tests performed on single copings and dental bridge models, although confirming the influence of the most crucial factors (i.e., abutment height and shape).

Within the limitations of the study, the authors draw the following conclusions:

(1) The number of impulses, performed on the bridge with a clinical removal tool, is a more reliable index of retrievability, compared to the measured force.
(2) The abutment height and taper angle have a higher influence on copings retentiveness than the luting agent.
(3) The best compromise for stability and retrievability consists of a long, slightly tapered abutment, cemented with a temporary cement.

## Figures and Tables

**Table 1:** Tested dental bridge models: height (mm) and taper angle (°) of each abutment.

Bridge	Abutment 1 Geometry	Abutment 2 Geometry
1	5 mm-0°	5 mm-0°
2	7 mm-0°	7 mm-0°
3	7 mm-2°	7 mm-2°
4	5 mm-2°	5 mm-4°
5	5 mm-0°	5 mm-4°
6	7 mm-2°	7 mm-4°
7	7 mm-0°	7 mm-4°
